# *Zingiber baumii* Chatan & Promprom: A New Species of Zingiberaceae From Thailand

**DOI:** 10.1002/ece3.70449

**Published:** 2024-10-17

**Authors:** Wilawan Promprom, Phukphon Munglue, Wannachai Chatan

**Affiliations:** ^1^ Department of Biology, Faculty of Science Mahasarakham University Maha Sarakham Thailand; ^2^ Plant and Innovation Research Unit Mahasarakham University Maha Sarakham Thailand; ^3^ Program of Biology, Faculty of Science Ubon Ratchathani Rajabhat University Ubon Ratchathani Thailand

**Keywords:** conservation assessment, dry dipterocarp forest, Endangered species, flowering plant, northeastern Thailand, plant diversity

## Abstract

*Zingiber baumii* Chatan & Promprom, a new species from the Zingiberaceae family, is described from Sakon Nakhon Province in northeastern Thailand. This species was identified during field expeditions conducted between 2018 and 2024 in Phu Pha Yol National Park. The morphological characteristics of *Z. baumii*, such as red or pinkish‐red leaf sheaths, leaf blades (1.5−) 2.0–3.0 cm wide, pink petioles, and bright yellow corolla with a red patch, distinguish it from closely related species such as *Z. isanense* Triboun & K. Larsen. *Zingiber baumii* grows in slightly shaded dry dipterocarp forests at elevations of 300–380 m. The preliminary conservation assessment indicates that this species should be classified as Endangered (EN) according to IUCN criteria due to its limited distribution and small population size. Detailed morphological comparisons with related species are provided, along with illustrations and habitat descriptions.

## Graphical abstract

*Zingiber baumii* Chatan & Promprom is a new species from the Zingiberaceae from Sakon Nakhon Province in northeastern Thailand. This species was found during field expeditions in Phu Pha Yol National Park.

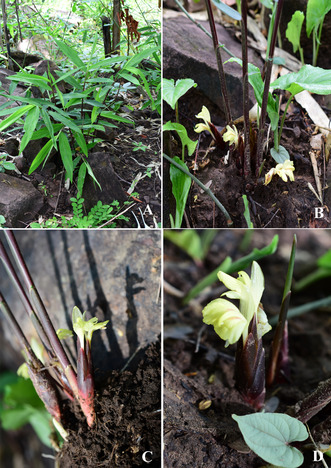

## 1 Introduction

The genus *Zingiber* Miller (1754: unpaged) in the family Zingiberaceae comprises approximately 180–210 species and is distributed from India to Malesia and extending to the western Pacific islands (Theerakulpisut et al. 2012; Tanaka and Aung 2017; POWO 2024), with its diversity center in Southeast Asia (Theerakulpisut et al. 2012; Wang 2000; Wu and Larsen 2000). *Zingiber* is considered one of the most widespread genera in the Zingiberaceae family (Triboun, Larsen, and Chantaranothai 2014). Morphologically, *Zingiber* consists of perennial herbs characterized by a pulvinate leaf base (i.e., a swollen part of the petiole) and a horn‐shaped anther crest embracing the upper part of the style (Bai, Leong‐Škorničková, and Xia 2015). The genus is divided into four sections based on the position of the inflorescence: *Zingiber* sect. *Zingiber*, sect. *Dymczewiczia* (Horan.) Benth., sect. *Pleuranthesis* Benth., and sect. *Cryptanthium* Horan. (Schumann 1904; Theerakulpisut et al. 2012; Bai, Leong‐Škorničková, and Xia 2015). In the sections *Dymczewiczia*, *Pleuranthesis*, and *Zingiber*, most species have spherical pollen with cerebroid or reticulate sculpturing, while those in section *Cryptanthium*, they have ellipsoidal pollen with spiro‐striate sculpturing (Liang 1988; Theilade et al. 1993; Triboun 2006; and Wu, Liao, and Wu 1996). Additionally, Thailand is known for its rich diversity of *Zingiber*, with about 56 to 59 species (Triboun, Larsen, and Chantaranothai 2014; Sangvirotjanapat and Newman 2023; POWO 2024).

The authors are interested in surveying plant diversity in Sakon Nakhon Province, northeastern Thailand, due to its high plant diversity. Recently, a new *Alocasia* species was discovered in this region (Promprom, Munglue, and Chatan 2024a) and a new subspecies of *Ardisia crenata* Sims (Promprom, Munglue, and Chatan 2024b). During expeditions to collect flowering plants in Sakon Nakhon Province, northeastern Thailand, between 2018 and 2024, some *Zingiber* specimens were collected. After carefully studying the morphology of the living plants and herbarium specimens and comparing them with other *Zingiber* species, the authors concluded that these specimens could not be referred to any previously named *Zingiber* species. Consequently, a new species, *Zingiber baumii*, is described here.

## 2 Materials and Methods

Specimens of an unknown *Zingiber* species were collected from a dry dipterocarp forest in Phu Pha Yol National Park, Sakon Nakhon Province, Thailand, in 2019. Morphological characters were examined based on herbarium specimens and type specimens deposited in BK and BKF, as well as living plants from the natural habitat. Morphological measurements were taken using a ruler, a vernier caliper, or an ocular micrometer under a dissecting microscope. The authors also consulted relevant taxonomic literature (e.g., Holttum 1950; Theilade 1996, 1999; Wu and Larsen 2000; Triboun, Larsen, and Chantaranothai 2014; Lê et al. 2019; Li et al. 2020; Wang, Lin, and Tseng 2020; Jayakrishnan et al. 2021; Sangvirotjanapat and Newman 2023). The preliminary conservation status of the new species was evaluated using field information and applying the criteria given by the IUCN Standards and Petitions Committee (2024).

## 3 Taxonomic Treatment

***Zingiber baumii* Chatan & Promprom, sp. nov**.

**FIGURE 1:**
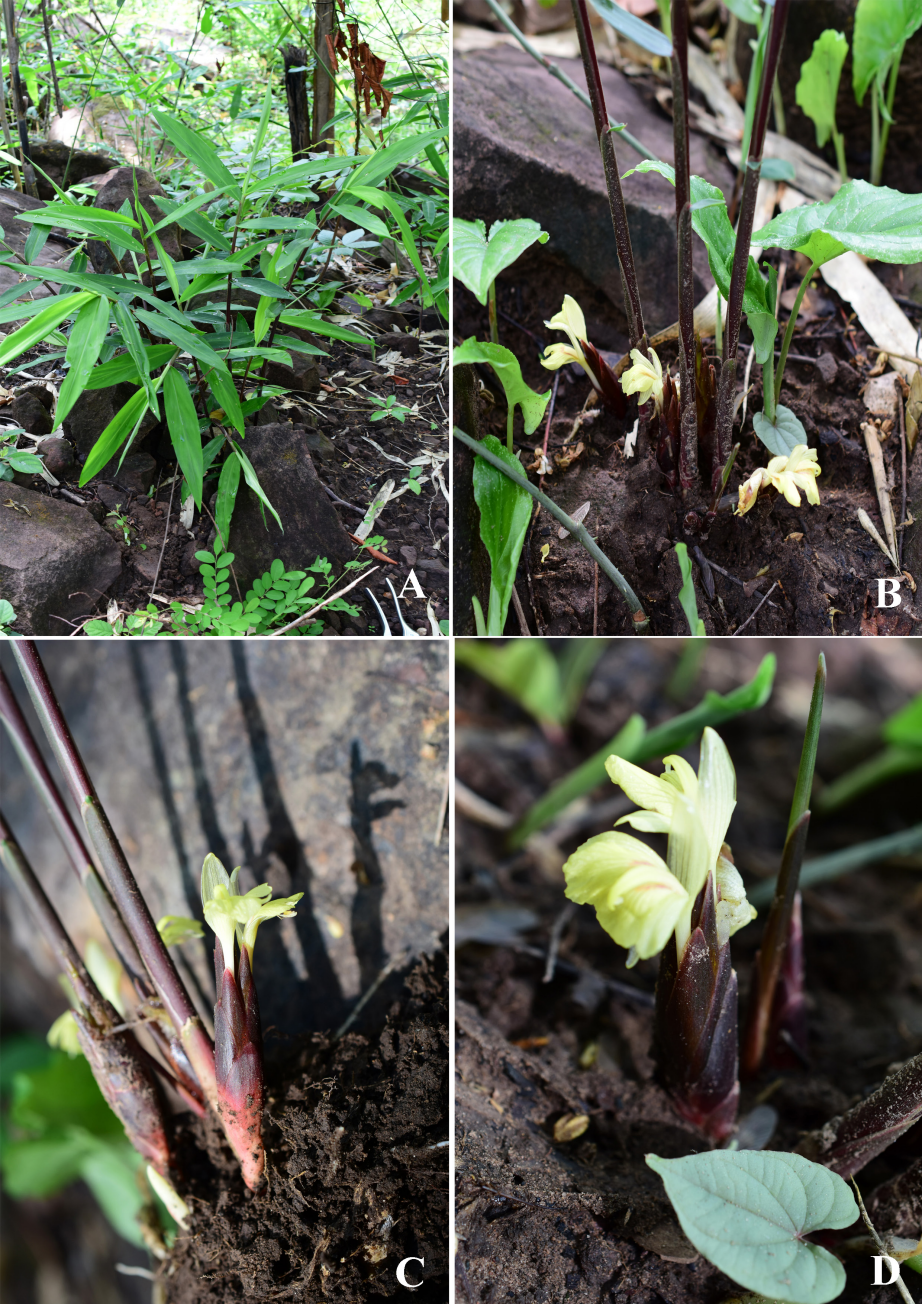
*Zingiber baumii* Chatan & Promprom (A) habit and habitat; (B) pseudostem and a group of inflorescences in natural habit; (C) inflorescence pulled from the ground; (D) one inflorescence (zoom out) and bracts, corolla, and staminodes. Photographed by Wilawan Promprom.

**FIGURE 2:**
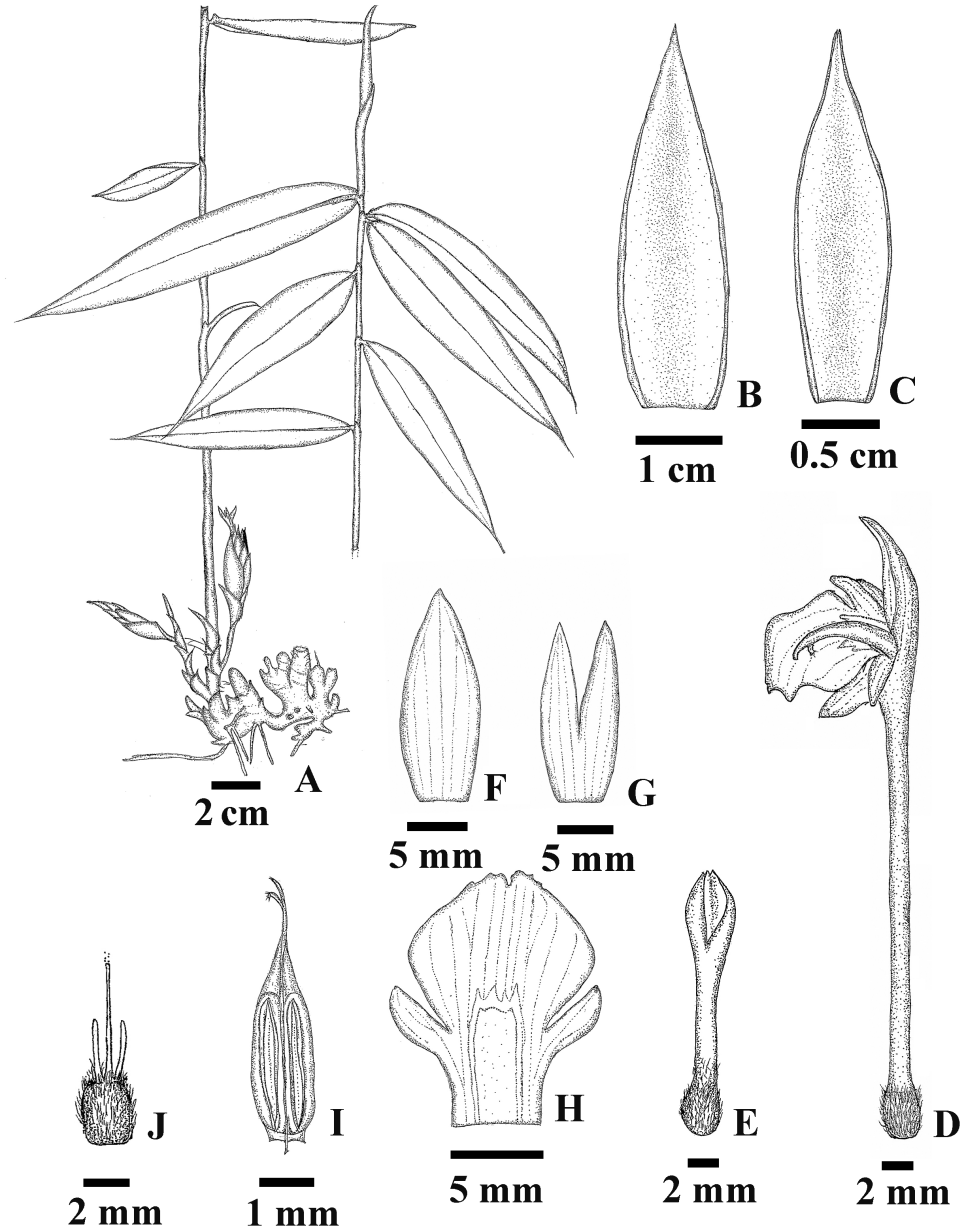
*Zingiber baumii* Chatan & Promprom (A) Habit, rhizome, pseudostem, leaves, and inflorescences; (B) bract; (C) bracteole; (D) floret; (E) ovary and calyx; (F) dorsal lobe of corolla; (G) lateral lobes of corolla; (H) labellum and side lobes; (I) anther; (J) ovary and stylode. Drawn by Wannachai Chatan from the type specimen (W. Chatan 2879).

Type:—Thailand. Sakon Nakhon Province: Phu Pha Yol National Park, 300–380 m alt., 16°58′37.9″ N 104°17′13.8″ E, 23 July 2019, *W. Chatan 2879* (holotype: BKF!; isotype: BK!) (Figures 1 and 2).

### 3.1 Diagnosis

Based on its morphology, *Zingiber baumii* is most similar to *Z. isanense* Triboun & K. Larsen as described in Triboun, Larsen, and Chantaranothai (2014), but it has distinct morphological differences from *Z. isanense* as follows: *Z. baumii* has a rhizome without a malodorous (bug‐like) smell when crushed, larger leafy shoots (4.5–5.2 mm in diameter), leaf sheaths that are always red or pinkish‐red in the lower part and purplish‐red or blackish‐red in the upper part, lanceolate leaf blades with (1.5−) 2.0–3.0 cm wide that are sparsely hairy on the abaxial surface, narrowly ovoid or ellipsoid spikes, bracts that are pale pink to moderately pink with purplish‐red to black‐red at the upper part, and have an acute or acuminate apex. Additionally, *Z. baumii* features a labellum that is always bright yellow with red patches, a corolla that is always bright yellow, and all staminodes are also bright yellow. The flowering period occurs in the middle of the rainy season, from July to August.

### 3.2 Description

*Rhizome* vertical, 2–3 cm long, leafy shoots close together; root tubers ovoid or narrowly ovoid, ca. 1.5–1.8 cm long. *Leafy shoot* 30–60 cm high, 4.5–5.2 mm in diameter, the lower 2–3 sheaths without leaf blades, 5–10 sheaths with leaf blades. *Leaf sheaths* red or pinkish‐red in the lower part, and red, purplish‐red, or blackish‐red in the upper part. *Leaf blade* lanceolate or narrowly lanceolate, 3.5–17.0 cm long, (1.5−) 2.0–3.0 cm wide, apex caudate or narrowly acute or rarely acute, base acute, green and glabrous above, pale green with sparsely hairy below, with a bug‐like smell when crushed. *Petiole* 1–2 mm long, pink or pinkish‐red, hairy on both surfaces. *Ligule* bilobed, 2.8–3.0 mm long and ca. 5.5–6.0 mm wide; each lobe ca. 3 mm wide, apex rounded, glabrous inside, sparsely hairs outside. Inflorescences 1–4, radical or lateral from rhizome. *Peduncle* erect, 1.3–1.5 cm long, pale pink or reddish pink with 2–3 sheathing bracts; the bracts slightly ovate, about 1.5–2.0 × 0.8–1.0 cm, pale pink or reddish. *Spike* narrowly ovoid or narrowly ellipsoid, more or less flat‐topped, apex slightly narrowly acute, 3.8–4.2 × 1.3–1.6 cm with 4–6 imbricate floral bract, distal bracts slightly in the same level as proximal bracts. *Floret* opening one time about in the afternoon, 1–2 at a time. *Bract* ovate or lanceolate, 4–5 × 0.9–1.0 cm, apex acute or acuminate with short mucro, pale pink to moderately pink on lower part and purplish‐red to black‐red on upper part, glabrous on adaxial side and sparsely haired on abaxial side. *Bracteole* lanceolate, 2.1–2.5 × 0.4–0.8 cm, white with small red dots near apex, translucent, apex narrowly acute and sometimes retuse, glabrous on adaxial side and sparsely haired on abaxial. *Calyx* white, translucent, 10–15 mm long, lower part tubular and upper part cone‐shaped, tube ca. 10 mm long and lobes ca. 5 mm long, glabrous on both surfaces except for few hairs on abaxial side near the tube base. *Corolla* bright yellow; tube 25–27 mm long, 1 mm diam., lobes lanceolate, apex acute or obtuse, glabrous on both surfaces; dorsal lobe 18–20 × 4.5–5.0 mm; lateral lobes 16–17 × 3.5–3.8 mm, base joined. *Staminode*: labellum bright yellow with red patch at center to base; midlobe broadly ovate, ca.10 × 8–9 mm, retuse at apex, margin irregular wavy; side lobes ovate or obovate or elliptic 5.5–6.0 × 2.5–2.8 mm, apex obtuse. *Filament* ca. 0.5–1 mm long. *Anther* ca 7.5 × 1 mm, yellow; anther crest 5–10 mm long, yellow, higher than stigma ca. 1 mm. *Ovary* broadly ovate, ca. 3.0 × 1.5 mm, densely haired on surface; epigynous glands ca. 4–5 mm long; ovule 6–10 per locule. *Epigynous glands* 2, filiform, ca. 5 mm long. *Fruit* ovoid or ellipsoid, 14–15 × 30–33 mm, greenish or greenish with some purplish red part, sparsely hairs on whole or slightly on whole surface. *Seeds* 4–5 per locule, ovoid, 5.0–5.2 × 9–9.5 mm, shine with a purple‐red color, glabrous, enveloped by the aril. Aril white, enveloping 2/3 of the length of the seeds or the whole seed length.

### 3.3 Additional Specimens Examined

*Zingiber baumii*: Thailand. Sakon Nakhon Province: Phu Pha Yol National Park, 300–380 m alt., 16°53′52.7″ N 104°02′58.8″ E, 12 August 2018, *W. Chatan 2697* (Paratype: BKF).

### 3.4 Phenology

The flowering period is in the middle of the rainy season (July to August), and the fruiting period is from August to September.

### 3.5 Distribution

**FIGURE 3:**
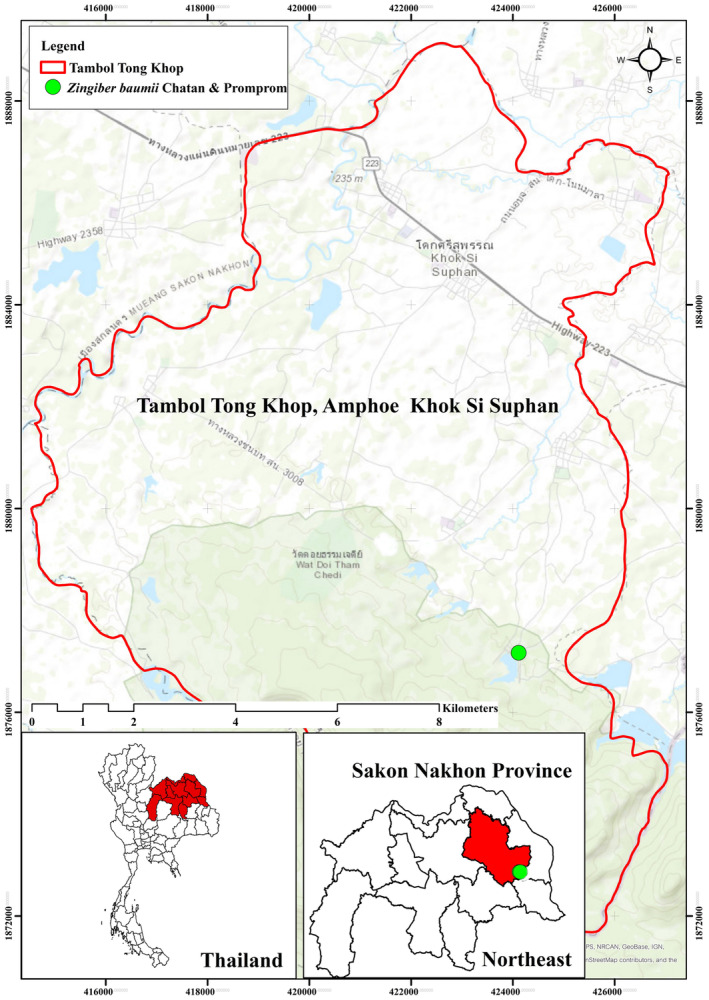
Distribution of *Zingiber baumii* Chatan & Promprom in Sakon Nakhon Province, Thailand (shown by green dot).

The new *Zingiber* species is found exclusively at its type locality, Phu Pha Yol National Park, Sakon Nakhon Province, in northeastern Thailand. It is endemic to Thailand (Figure 3).

### 3.6 Ecology

This new species grows in slightly shaded areas of dry dipterocarp forests at an elevation of 300–380 m.

### 3.7 Vernacular Name

Khing Pa Lueang.

### 3.8 Etymology

The specific epithet of *Zingiber baumii* refers to Dr. Bernard R. Baum, my coadvisor during my PhD studies, from Agriculture and Agri‐Food Canada, Eastern Cereal and Oilseed Research Centre, Ottawa, Ontario, Canada. He is an expert in both classical and advanced plant taxonomy. He has dedicated his entire life to the field of plant taxonomy.

### 3.9 Preliminary Conservation Status

The population of the new species was found only in one place in the type locality in Thailand. The plant is estimated to number fewer than 2500 mature individuals and mature individuals in each subpopulation < 250. Therefore, it should be considered as “Endangers (EN),” according to IUCN criteria C(C2ai) (IUCN Standards and Petitions Committee 2024).

## 4 Discussion

The inflorescence of *Zingiber baumii* is borne on erect peducle, so it belong to section *Zingiber* (Schumann 1904; Theerakulpisut et al. 2012; Bai, Leong‐Škorničková, and Xia 2015). Its morphological characters are very similar to *Z. isanense*, the plant previously named from Thailand and Laos (Triboun, Larsen, and Chantaranothai 2014). Both *Z. baumii* and *Z. isanense* exhibit similar morphological characteristics, such as having rhizomatous herb with vertical rhizomes and clump‐forming growth habits. They both possess leafy shoots that reach heights of 30–60 cm, with multiple sheaths, although the specific diameters of the shoots differ. Their leaf blades are green and glabrous on the upper surface, with varying degrees of hairiness on the abaxial side. Additionally, both species feature bilobed ligules, radical and erect inflorescences originating directly from the rhizome, and peduncles that bear sheathing bracts. Their bracteoles are white and display varying levels of hairiness. The calyx tubes of both species are glabrous with some hairs, and their corolla tubes are similar in length but differ in color. Both species produce ovoid or ellipsoid fruits containing seeds that are enveloped by an aril, highlighting their close morphological relationship within the genus *Zingiber*.

*Zingiber baumii* and *Z. isanense* exhibit several distinct morphological differences. *Z. baumii* has a rhizome without a malodorous (bug‐like) smell when crushed, whereas *Z. isanense*'*s* rhizome emits this smell. The leafy shoots of *Z. baumii* are larger in diameter (4.5–5.2 mm) compared with *Z. isanense* (2.8–3.5 mm). Their leaf sheaths differ in color, with *Z. baumii* showing red or pinkish‐red in the lower part and purplish‐red or blackish‐red in the upper part, while *Z. isanense* displays green or purple in the lower part. The leaf blades of *Z. baumii* are lanceolate and sparsely hairy on the abaxial surface, contrasting with the linear, grass‐like, glabrous, or glabrescent leaves of *Z. isanense*. Additionally, the spikes of *Z. baumii* are narrowly ovoid or ellipsoid, while those of *Z. isanense* are capitulate. *Z. baumii*'*s* bracts are pale pink to moderately pink with purplish‐red to black‐red at the upper part and an acute or acuminate apex, whereas *Z. isanense*'*s* bracts are whitish green or greenish purple with purplish dots and a long acuminate or caudate apex. The flowers of *Z. baumii* are bright yellow with red patches, while those of *Z. isanense* are white with yellow near the base. The flowering period of *Z. baumii* is in the middle of the rainy season (July to August) with fruiting from August to September, whereas *Z. isanense* flowers before the rainy season (late May to June).

The details of the morphological differences between *Z. baumii* and Z. *isanense* are presented in Table 1.

**TABLE 1:** Comparison of the different morphological characters between *Zingiber baumii* Chatan & Promprom and *Z. isanense* Triboun & K. Larsen.

Characters	*Zingiber baumii*	*Zingiber isanense*
Rhizome	Absence of malodorous (bug‐like) smell when crushed	Presence of malodorous (bug‐like) smell when crushed
Leafy shoot	4.5–5.2 mm diameter	2.8–3.5 mm in diameter
Leaf sheath	Red or pinkish‐red in the lower part, and red, purplish‐red, or blackish‐red in the upper part	Green or purple in the lower part
Leaf blade	Lanceolate or narrowly lanceolate, 3.5–17.0 cm long, (1.5−) 2–3.0 cm wide, apex caudate or narrowly acute or rarely acute, sparsely hairy on abaxial surface	Linear, grass‐like, 9–16 by 1–1.7 cm, apex very long and narrowly acute, glabrous or glabrescent on abaxial surface
Petiole	1–2 mm long, pink or pinkish‐red	2–3 mm long, light green with some red dots
Peduncle	1.3–1.5 cm long, pale pink or reddish pink pale pink or reddish sheathing bracts	5–10 cm long, with white, pale pink, or purple sheath bracts
Spike	Narrowly ovoid or narrowly ellipsoid, more or less flat‐topped, apex slightly narrowly acute	Capitulate, 3–4 by 1–1.5 cm, apex narrowly acuminate
Bract	4–5 × 0.9–1.0 cm, pale pink to moderately pink on the lower part and purplish‐red to black‐red on the upper part, with an acute or acuminate apex and a short mucro	Approximately 2.7 × 0.7 cm, whitish green or greenish purple with purplish dots near the apex, apex long acuminate or caudate
Bracteole	White with small red dots near apex, translucent, apex narrowly acute and sometimes retuse	White, apex acute, glabrescent to pubescent
Calyx tube	ca. 10 mm long and lobes ca. 5 mm long, glabrous on both surfaces except for few hairs on abaxial side near the tube base	Calyx tube ca. 5 mm long, lobes 8–9 mm long, apex hairy
Corolla tube	Always bright yellow, never white, 25–27 mm long, dorsal corolla lobe 18–20 × 4.5–5.0 mm, lateral lobes 16–17 × 3.5–3.8 mm	White, 32–33 mm long, dorsal corolla lobe 14–16 × 4–5 mm, lateral corolla lobes 12–14 by 3–4 mm
Labellum	Always clear bright yellow with red patch at center to base; midlobe broadly ovate, ca. 10 × 8–9 mm	White, yellow near base, midlobe rounded, 10–13 × 10–13 mm
Filament	ca. 0.5–1 mm long.	Filament ca. 2 mm long
Anther	ca. 7.5 × 1 mm, yellow	6–7 × 2–3 mm, cream‐yellow
Ovary	Densely haired on the whole surface	Glabrescent or pubescent in upper half
Fruit	Greenish or greenish with some purplish red part, sparsely hairs on whole or slightly on whole surface	Pinkish to light red, surface glabrescent
Seed	4–5 per locule	3–4 per locule
Phenology	Flowering period is in the middle of the rainy season (July to August), and the fruiting period is from August to September	Flowering period is before the rainy season (late May to June)

## Author Contributions

**Wilawan Promprom:** data curation (equal), formal analysis (equal), funding acquisition (equal), investigation (equal), methodology (equal), project administration (equal), resources (equal), validation (equal), writing – original draft (equal). **Phukphon Munglue:** data curation (equal), formal analysis (equal), investigation (equal), methodology (equal), resources (equal), validation (equal), visualization (equal). **Wannachai Chatan:** conceptualization (lead), data curation (equal), formal analysis (equal), funding acquisition (equal), investigation (equal), methodology (equal), project administration (equal), resources (equal), supervision (equal), visualization (equal), writing – original draft (equal), writing – review and editing (equal).

## Conflicts of Interest

The authors declare no conflicts of interest.

## Data Availability

The authors have nothing to report.
